# Spatiotemporal Variations and Driving Factors of Air Pollution in China

**DOI:** 10.3390/ijerph14121538

**Published:** 2017-12-08

**Authors:** Dongsheng Zhan, Mei-Po Kwan, Wenzhong Zhang, Shaojian Wang, Jianhui Yu

**Affiliations:** 1 Key Laboratory of Regional Sustainable Development Modeling, Institute of Geographic Sciences and Natural Resources Research, Chinese Academy of Sciences, Beijing 100101, China; zhands@126.com (D.Z.); yujh@igsnrr.ac.cn (J.Y.); 2 University of Chinese Academy of Sciences, Beijing 100049, China; 3 Department of Geography and Geographic Information Science, University of Illinois at Urbana-Champaign, Urbana, IL 61801, USA; mpk654@gmail.com; 4 Department of Human Geography and Spatial Planning, Utrecht University, P.O. Box 80125, 3508 TC Utrecht, The Netherlands; 5 Guangdong Provincial Key Laboratory of Urbanization and Geo-simulation, School of Geography and Planning, Sun Yat-sen University, Guangzhou 510275, China; 1987wangshaojian@163.com

**Keywords:** air pollution, spatiotemporal variations, driving factors, geographical detector, China

## Abstract

In recent years, severe and persistent air pollution episodes in China have drawn wide public concern. Based on ground monitoring air quality data collected in 2015 in Chinese cities above the prefectural level, this study identifies the spatiotemporal variations of air pollution and its associated driving factors in China using descriptive statistics and geographical detector methods. The results show that the average air pollution ratio and continuous air pollution ratio across Chinese cities in 2015 were 23.1 ± 16.9% and 16.2 ± 14.8%. The highest levels of air pollution ratio and continuous air pollution ratio were observed in northern China, especially in the Bohai Rim region and Xinjiang province, and the lowest levels were found in southern China. The average and maximum levels of continuous air pollution show distinct spatial variations when compared with those of the continuous air pollution ratio. Monthly changes in both air pollution ratio and continuous air pollution ratio have a U-shaped variation, indicating that the highest levels of air pollution occurred in winter and the lowest levels happened in summer. The results of the geographical detector model further reveal that the effect intensity of natural factors on the spatial disparity of the air pollution ratio is greater than that of human-related factors. Specifically, among natural factors, the annual average temperature, land relief, and relative humidity have the greatest and most significant negative effects on the air pollution ratio, whereas human factors such as population density, the number of vehicles, and Gross Domestic Product (GDP) witness the strongest and most significant positive effects on air pollution ratio.

## 1. Introduction

Due to rapid industrialization and urbanization over the past few decades, many developing countries, especially the largest ones like China, are experiencing serious air pollution along similar historical trajectories as developed countries [1,2,3,4]. Considerable evidence has shown that air pollution is linked to a wide range of adverse effects, including harmful health outcomes such as increased mortality and morbidity [5,6], economic loss in urban areas, and ecological damage [7,8]. According to the World Health Organization (WHO), ambient air pollution caused 3 million premature deaths worldwide in 2012, of which over 1 million deaths were contributed by China [9]. Further, the Asian Development Bank pointed out that less than 1% of the 500 largest cities in China meet the air quality standards recommended by WHO, with 7 cities ranked among the 10 most polluted cities in the world [10]. Therefore, accurate identification of the spatiotemporal variations of air pollution in China and its associated driving factors is of great significance for designing appropriate policies for atmospheric pollution control and abatement.

With growing public awareness of the detrimental effects of air pollution, extensive empirical studies have focused overwhelmingly on the spatiotemporal variations of air pollution that are imperative to evaluate the health risks of human exposure. The spatial characteristics of air pollution have been examined at different scales in previous studies, ranging from local scale to national scale [1,11,12,13]. Given the limited number of ground monitoring stations across Chinese cities to date, the majority of extant studies conducted in China focus largely on relatively small areas or regions, concerning major metropolitan cities such as Beijing, Shanghai, and Guangzhou [14,15,16] and the urban agglomerations of Jing-Jin-Ji [12], Bohai Rim [17], and Yangtze River Delta [13]. However, due to the lack of complete air quality data, the spatiotemporal variations of air pollution in China at the national scale based on ground monitoring data are, to date, not fully understood.

Recently, some emerging studies have provided insights into air pollution at the national scale using spatial interpolation methods based on data collected at available sample sites [18] or remote sensing interpretation of Aerosol Optical Depth (AOD) [11,19]. However, the accuracy of pollutant concentration estimates using these methods is affected by many factors, including sample size, the density of ground monitoring stations [18,20], the spatial resolution of remote sensing images [19], and the complexity of the transformation coefficient between AOD and air pollutant concentration [21]. Data collected by ground monitoring stations may provide much more precise reported air pollution values. However, few studies have used these data due to a general lack or sparse coverage of this type of data. Alternatively, to improve the accuracy of pollutant concentration estimates, land use regression (LUR) models have been used to estimate air pollution levels at fine spatial scales, with several auxiliary variables such as land cover, road type, traffic volume, and distance to pollution sources [22]. Likewise, LUR models are also limited in their ability to predict the level of air pollution at a national scale. On the other hand, although continuous air pollution episodes in China have frequently been reported by local and foreign media, and were found to be associated with increased risk of hospital outpatient visits or admissions relative to the period of nonconcentrated air pollution [23,24], past studies have mostly considered air pollution as a static process while ignoring the persistence of air pollution from a dynamic perspective [25]. Hence, empirical evidence on continuous air pollution is still lacking in China.

To better understand the driving factors of air pollution, much research from the micro perspective has focused on source apportionment and chemical composition of ambient air pollution [26,27]. This strand of research has revealed the contributions of different emission sources or regions to local air pollution but failed to explain the spatial disparity in air pollution levels in different cities. It thus cannot inform regional and national air pollution mitigation planning. From the macro perspective, a large body of studies has identified the associations between air pollution levels and various human-related factors, including urbanization rate, population, population density, GDP, per capita GDP, industrial structure, energy consumption, and the number of vehicles [28,29,30,31]. Among natural factors, land use type, elevation, Normalized Difference Vegetation Index (NDVI), and meteorological conditions [18,32] have effects on air pollution that have also been well documented in the literature. Nevertheless, most studies conducted in China have investigated the influence of either natural or human factors on air pollution separately, and few studies have considered the combined effects of both natural and human factors on air pollution at the national scale except for a recent study by Luo et al. [33]. Another limitation is that previous studies have overlooked the magnitude or effect intensity of the driving factors on air pollution. Consequently, from a methodological point of view, some innovative approaches are needed to address which driving factors are more significant predictors of air pollution in China.

Considering frequent air pollution episodes in China, especially in recent years, The Chinese Government has also attached great importance to controlling and alleviating air pollution through a series of regulatory policies, such as the third National Ambient Air Quality Standard issued in February 2012 and the Ten Specific Measures of Air Pollution Prevention and Control Action Plan published in September 2013. More recently, Premier Keqiang Li has made an unequivocal commitment in the 2016 government work report that the ratio of days with good air quality should exceed 80% in all cities above the prefectural level in China during the period of the 13th Five Year Plan (2016–2020). In other words, the ratio of days with air pollution in cities above the prefectural level in China should stay below 20% by 2020. Given the above knowledge gaps and Chinese government’s policy goals, this paper examines the spatiotemporal variations of air pollution in China and its associations with various driving factors, using air quality data collected by ground monitoring stations in 2015 in Chinese cities above the prefectural level. Specifically, the present study seeks to (1) investigate the spatial and temporal variations of the air pollution ratio and continuous air pollution ratio in China at the national scale, and (2) determine the effect intensity and direction of both natural and human factors on the air pollution ratio in China using the geographical detector model and Pearson correlation analysis.

## 2. Study Area, Data, and Methods

### 2.1. Study Area

China is the largest developing country in the world. It has the world’s largest population of 1.37 billion, the second-largest Gross Domestic Product (GDP) of 68,905.2 billion RMB in 2015, and the third-largest land area, with a complex geography and tremendous socioeconomic disparities within the country. Due to the availability and integrity of both air quality data and data of relevant driving factors of air pollution, the study area for this research is mainland China, including all 335 cities above the prefectural level and another 3 county-level cities (i.e., Korla, Shihezi, and Wujiaqu) that are under the direct jurisdiction of Xinjiang province. Hereafter, this paper refers to these cities simply as “the selected cities” or “these cities.” Note that Hong Kong, Macao, Taiwan, and other county-level cities under the direct jurisdiction of Hubei and Hainan provinces or their prefecture-level cities were excluded from this study due to the lack of complete data.

### 2.2. Data Sources

#### 2.2.1. Air Pollution Data

To improve the accuracy of air quality measurement and strengthen air pollution control in China, the Ministry of Environmental Protection of China released the new National Ambient Air Quality Standard (GB3095-2012) in February 2012. This was the first attempt to monitor real-time PM_2.5_ (the fine particulate matters under 2.5 μm) and 8-h O_3_ concentration in China. However, it was not until 2015 that ground air quality monitoring stations in all cities above the prefectural level in China were completely established. Considering data integrity, this study used ground monitoring air quality data for the whole year of 2015 obtained from the Ministry of Environmental Protection of the China (http://datacenter.mep.gov.cn/index) and selected the year 2015 as the study period. This air quality dataset consists of the names of the monitoring cities, daily record dates, values of the daily Air Quality Index (AQI) (which measures air quality), and primary pollutants in detail. Note that there are some missing data for some cities due to power failure, routine maintenance, instrument trouble, or communication failure. Because these missing data account for only a small percentage of all data ranging from 0.55% to 9.32% and are nearly randomly distributed, they were disregarded in our analysis and the remaining data can still reliably represent air pollution levels in the selected Chinese cities.

#### 2.2.2. Data of the Driving Factors of Air Pollution 

This study aimed to investigate the combined effect of both natural and human-related factors on air pollution levels in China. Given the theoretical frameworks used in previous research and the data availability, explanatory variables in this study consist of eight natural factor variables, including elevation (ELE), land relief (LR), annual average temperature (AAT), annual average precipitation (AAP), wind speed (WS), relative humidity (RH), sunshine hours (SH), and air pressure (AIRP). The human factors considered in this study consist of seven variables, including population (POP), population density (POPD), Gross Domestic Product (GDP), per capita GDP (PGDP), secondary industry ratio (SIR), the number of vehicles (NOV), and urbanization rate (UR).

Elevation data were obtained from a digital elevation model, with a spatial resolution of 90 m, provided by the Geospatial Data Cloud (http://www.gscloud.cn/). Combining these raster DEM data and the vector data of the boundaries of the selected Chinese cities, the average elevation for each city was obtained using the zonal statistics tool in ArcGIS 10.2 software (ESRI, Redlands, CA, USA). Additionally, land relief was calculated to reflect the range of elevation within a given area unit using Digital Elevation Model (DEM) data following the method in [34]. Meteorological condition data from January 2001 to December 2014 were derived from the Meteorological Data Center of the Chinese Meteorological Administration (http://data.cma.cn/), including monthly meteorological factors (i.e., temperature, precipitation, wind speed, relative humidity, sunshine duration, and air pressure) from 960 national meteorological stations. Based on the location of these meteorological stations, continuous meteorological surfaces across the whole study area were generated using ordinary kriging, which may generate more accurate meteorological estimates by cross-validation [35]. The average values of all selected meteorological factors for each city were then calculated using the zonal statistics tool based on the same approach used for the DEM data.

Given the availability of human factor data in the selected cities, selected human factor data were mainly drawn from the 2014 China Statistical Yearbook for Regional Economy, including population, land area, GDP, per capita GDP, secondary industry ratio, and the number of vehicles. The population density for each city was obtained by dividing the population of each city by its land area. In addition, the urbanization rate was derived from the urban population divided by the total population, which were both derived from the 2010 Population Census of China by County due to the lack of complete data in other years. Then, all human factor data were integrated and linked to the vector data of the boundaries of the selected cities using ArcGIS software (ESRI, Redlands, CA, USA). In the end, since the geographical detector model only handles categorical variables (similar to ANOVA), all continuous natural and human-related variables were transformed into five categorical variables using the quantile method in ArcGIS before running the model. The quantile method was chosen because it yielded more reasonable sample sizes in the subgroups when compared with the traditional natural breaks classification method.

### 2.3. Method: Air Pollution Measurement and Modeling

#### 2.3.1. Air Quality Measurement and Indexes 

Many indexes have been utilized to reflect the level of air pollution in previous studies, including individual pollutant concentration (e.g., PM_2.5_, PM_10_, SO_2_, NO_2_, O_3_) [36] and a widely used aggregate indicator named Air Quality Index (AQI) [37]. Among these indexes, the Air Quality Index is generally considered an appropriate index for representing overall urban air quality due to its comprehensiveness. The equations of the Air Quality Index are shown as follows:

(1) $$IAQI_{p}=\frac{IAQI_{Hi}-IAQI_{Lo}}{BP_{Hi}-BP_{Lo}}\left(C_{p}-BP_{Lo}\right)+IAQI_{Lo}$$

where $IAQI_{p}$ is an individual air quality index (IAQI) for pollutant P, including PM_2.5_, PM_10_, SO_2_, NO_2_, CO and O_3_; $C_{p}$ is the concentration of pollutant P; $BP_{Hi}$ and $BP_{Lo}$ are the limited value greater than or equal to $C_{p}$ and the limited value less than or equal to $C_{p}$, respectively; and $IAQI_{Hi}$ and $IAQI_{Lo}$ are the limited air quality indexes corresponding to $BP_{Hi}$ and $BP_{Lo}$.

On this basis, AQI is defined as the maximum value of the IAQI,

AQI = max{IAQI_1_, IAQI_2_, IAQI_3_,…, IAQI*_n_*} (2)

where AQI denotes the air quality index in a city. IAQI*_n_* is the individual air quality index for pollutant P, as mentioned above.

Table 1 presents the air quality categories for different pollutants based on different concentration levels for calculating the IAQI and AQI. As shown in the table, AQI is classified into six levels, types, and health effects. The value of the AQI ranges from 0 to 500. The higher the AQI level, the more serious the air pollution type and its health effects, and vice versa. In general, air quality is considered fine with AQI ≤ 100 and polluted when AQI > 100. In the rest of this paper, the values of the air pollution ratio and continuous air pollution ratio are derived based on this definition: days with AQI > 100 are called “days with air pollution” and days with AQI ≤ 100 are called “days with good air quality” or “days without air pollution” although the latter actually will have certain levels of air pollution.

With respect to the measurement of air quality, both the average AQI [37] and structural indexes, such as the ratio of days with AQI ≤ 100 (days with good air quality) [28] or the ratio of days with AQI > 100 (when air pollution types are classified as light to serious) (hereafter referred to as the air pollution ratio) [38], have been used to reflect the level of air pollution. Despite the high correlation between the average AQI and the air pollution ratio, the average AQI is susceptible to extreme values and fails to reflect the actual number of days with adverse or harmful levels of air pollution. Thus, since the air pollution ratio reflects not only the level but also the duration of air pollution, this study uses it as the measurement of air pollution levels. The equation of the air pollution ratio is shown as follows:

R = N_p_/N_o_ (3)

where R is the air pollution ratio, and N_p_ and N_o_ denote the number of days for which the AQI is higher than 100 (days with harmful levels of air pollution) and the number of total observed days in the specified period in a city, respectively.

**Figure 1:**
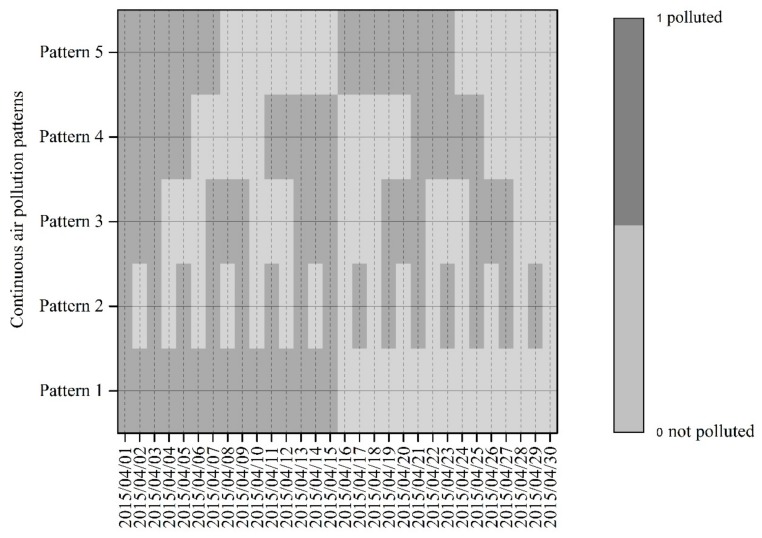
Hypothetical scenarios of continuous air pollution patterns in five cities. The figure assumes that the temporal distributions of daily air quality index in April 2015 for five Chinese cities have different continuous air pollution patterns.

In addition to the air pollution ratio, continuous air pollution (CAP) was another research emphasis in this study because of its substantial role in affecting health. Following previous studies carried out in China [39], continuous air pollution is defined as a persistent pollution episode with three or more consecutive days with air pollution (AQI > 100), and the continuous air pollution ratio is measured by the number of days with continuous air pollution divided by the total observed days (denoting each month or the whole year of 2015 in this study). Figure 1 presents five hypothetical temporal patterns of daily AQI values. In the figure, AQI was treated as a dichotomous variable where 1 indicates a day with air pollution (AQI > 100) and 0 indicates a day without air pollution (AQI ≤ 100). Despite having the same number of air pollution days (15 days) in the five patterns, there are significant differences in the times and the average and maximum values of continuous air pollution, where times of continuous air pollution from Pattern 1 to Pattern 5 are 1, 0, 5, 3, 2; the average values of continuous air pollution are 15, 0, 3, 5, 7.5; and the corresponding maximum values of continuous air pollution are 15, 0, 3, 5, 8. Based on epidemiological evidence on the positive association between hospital outpatient visits or admissions and the duration of continuous air pollution [23,24], it can be argued that Pattern 1 is likely to exert greater detrimental health effects than Pattern 2; this is one reason why the current study takes into account continuous air pollution.

#### 2.3.2. The Geographical Detector Model

In this study, the geographical detector model was employed to identify the effect intensity of geographical factors that influence air pollution in China. It is a spatial variation method that has been widely used to explore the relationship between environmental factors and human health outcomes [40,41]. The basic idea of the geographical detector method is that the dependent variable *Y* is closely related to the independent variables *X* if their spatial distributions tend to be consistent. Compared with other regression methods, the advantage of the geographical detector model is that there is no need to consider multicollinearity among the explanatory variables since their effect intensity is tested separately [42]. The geographical detector model comprises four parts (i.e., the factor detector, the ecological detector, the interaction detector, and the risk detector), among which the factor detector is a significant component which measures the power of determinant (PD). The equation of the factor detector is as follows [41]:

(4) $$\mathrm{PD}=1-\frac{1}{n\sigma^{2}}\sum_{h=1}^{L}n_{h}\sigma_{h}{}^{2}$$

where n and $\sigma^{2}$ denote the sample size and the variance of the air pollution ratio of the whole study area, respectively. According to spatial heterogeneity, the study area can be divided into *L* strata, denoted by *h* = 1, 2,…, *L*; $n_{h}$ and $\sigma_{h}{}^{2}$ refer to the sample size of each subregion and the variance of air pollution ratio for each subregion, respectively. The value of PD ranges from 0 to 1. If a determining factor completely controls the air pollution ratio, the value of PD is 1. If a determining factor is completely unrelated to the air pollution ratio, the value of PD is 0. The larger the value of PD, the greater the impact the determinant factor has on air pollution. The value of PD can therefore be used to explain the extent to which a determining factor exerts influence on the air pollution ratio.

## 3. Results

### 3.1. Descriptive Statistics of Air Pollution

Table 2 shows the descriptive statistics of several critical variables regarding air pollution in China. As shown in the table, the air pollution ratio in the 338 Chinese cities in 2015 ranges from 0% to 80.7%, with an average of 23.1 ± 16.9%, indicating that air pollution in China is serious, with the average number of days with air pollution exceeding a fifth of the year in 2015 and considerable spatial disparity in air pollution among the selected cities. According to the air pollution levels presented in Table 1, heavy-above air pollution (also called heavy smog or haze) ratio refers to the ratio of days with heavy or serious air pollution (AQI > 200) in the year. Although the average heavy-above air pollution ratio accounts only for 3.1% over the whole study area, the city with the maximum ratio has a ratio of 38.1%, a startling figure which indicates that this city had heavy-above air pollution over a third of the year.

As for continuous air pollution, the CAP ratio in 2015 in the selected cities varies spatially, with an average of 16.2 ± 14.8%. Moreover, the average times of CAP in these cities is 10.7 ± 8.8, and its maximum is 38 times in 2015, suggesting that Chinese cities tend to witness frequent occurrences of continuous air pollution episodes in the year. Again, the highest value of the maximum and the average CAP are 123 and 23, respectively, implying that certain Chinese cities experienced serious continuous air pollution with long duration in 2015. However, note that there are five cities (i.e., Tacheng, Aba, Ngari, Diqing, and Chuxiong) situated mainly in the Southwestern and Northwestern regions in China where air pollution was nonexistent in 2015, let alone continuous air pollution episodes.

### 3.2. Spatial Variations of Air Pollution

**Figure 2:**
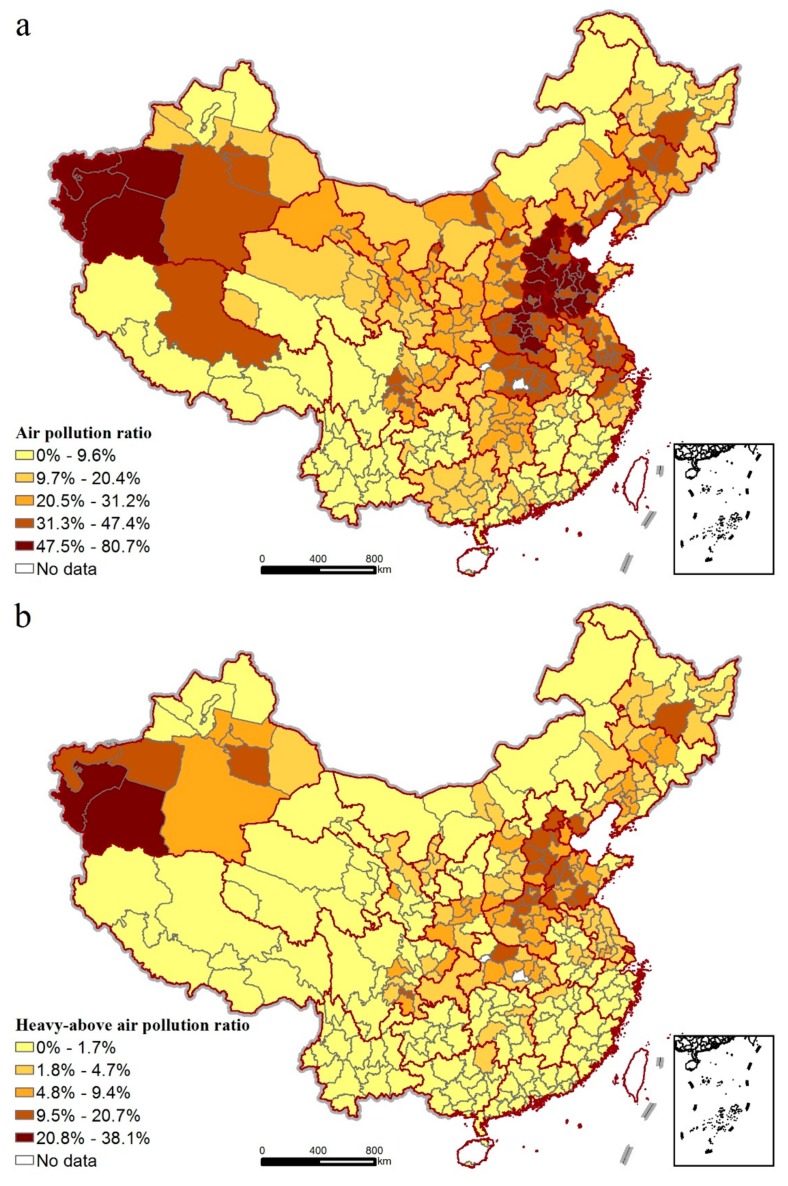
Spatial variations of air pollution ratio in China. (**a**) air pollution ratio (**b**) heavy-above air pollution ratio.

As illustrated in Figure 2a, the air pollution ratio is classified into five categories using the natural breaks classification method in ArcGIS, where the upper breakpoint of the second level was manually adjusted from 20.4% to 20.0% to reflect major nonattainment cities for policy goals. As shown in the figure, the air pollution ratio in China varies greatly across the whole study area, and 179 cities (or 46.1%) are recorded to have an air pollution ratio of more than 20%. Cities with the highest level of air pollution ratio (>47.5%) account for 9.8% of the total number of cities (33 cities out of 338 cities). They are distributed mainly in North China, especially in the Bohai Rim region and Xinjiang provinces, while cities with the lowest level (<9.6%) are located mainly in South China along with only a few cities in the northeastern and northwestern regions. This result reveals a distinct spatial disparity in air pollution level between North China and South China. Moreover, 58 cities (or 17.2%) located mostly in Hubei, Jiangsu, and Tibet provinces are also found to have relatively high levels of air pollution ratio ranging from 31.3% to 47.4%. As for the heavy-above air pollution ratio presented in Figure 2b, a pronounced spatially concentrated trend is observed when compared with the spatial variations of the air pollution ratio, where areas with a higher heavy-above air pollution ratio (>9.5%) are distributed largely over several cities, located in the Xinjiang, Heilongjiang, Henan, and Hubei provinces in addition to the Bohai Rim region.

**Figure 3:**
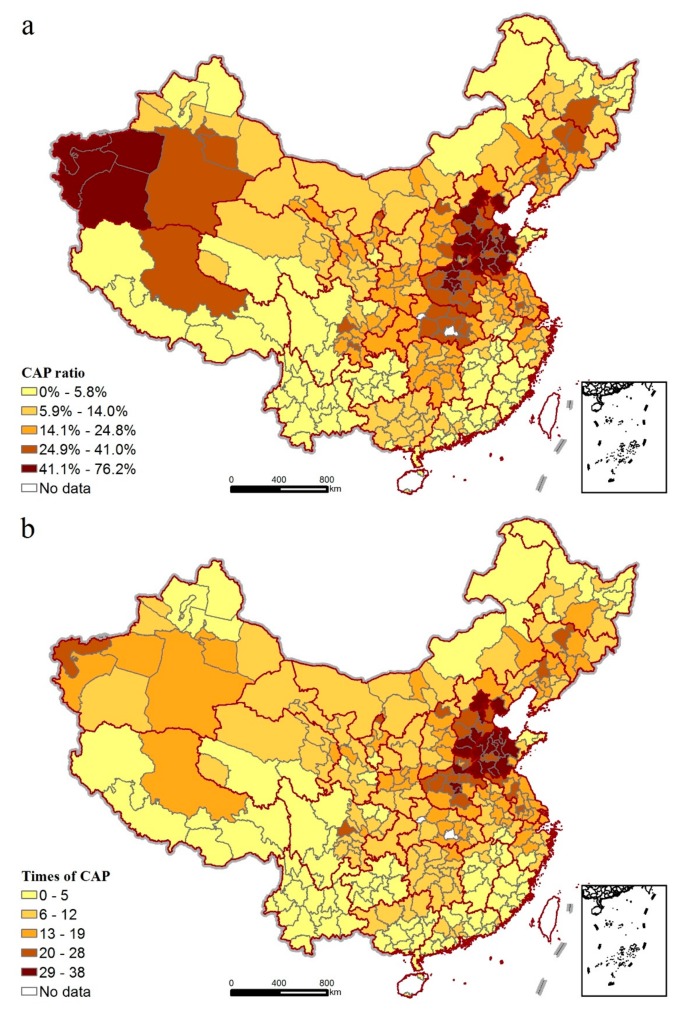

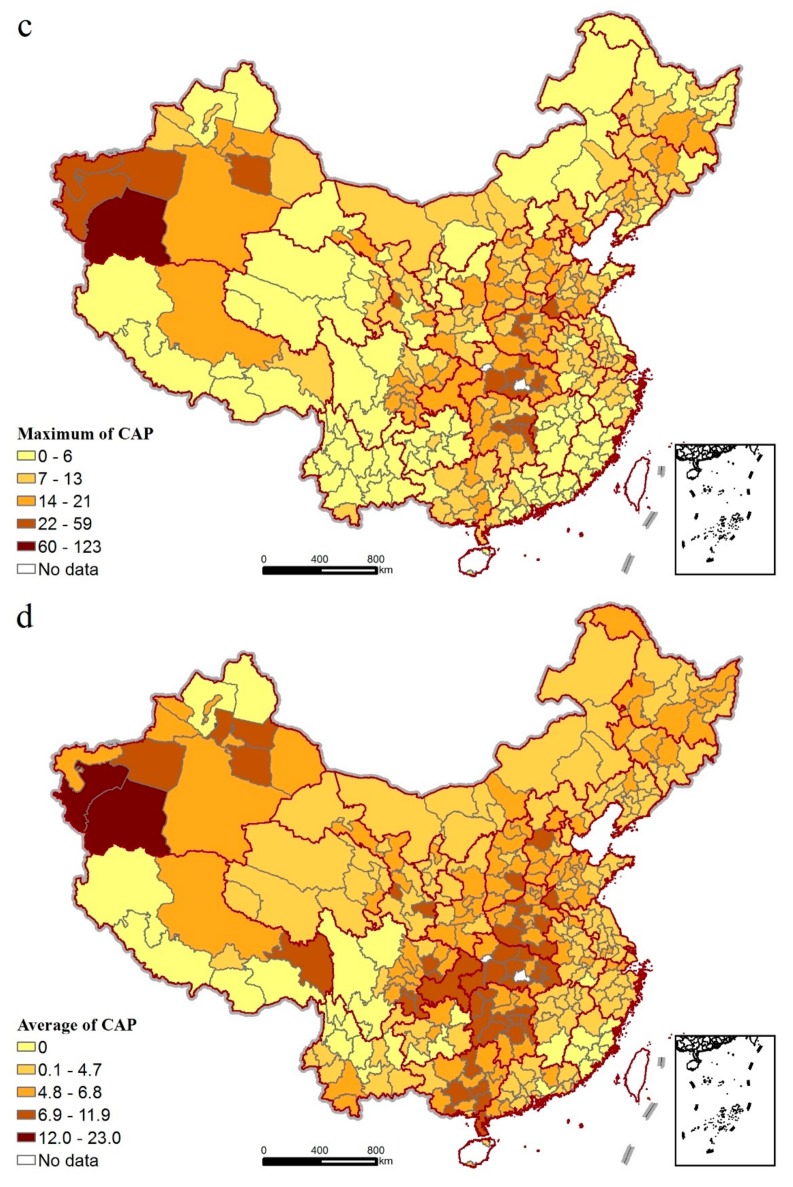
Spatial variations of continuous air pollution (CAP) in China. (**a**) CAP ratio (**b**) Times of CAP (**c**) Maximum of CAP (**d**) Average of CAP.

Regarding continuous air pollution, the spatial distribution of the CAP ratio depicted in Figure 3a presents a similar variation to that of the air pollution ratio, a finding implying that cities with elevated air pollution ratios are more likely to experience a high continuous air pollution ratio. Times of CAP, an index measuring how often a continuous air pollution episode occurs, shows a range from 0 to 38 over the study area, with cities suffering frequent continuous air pollution (more than 20 times) distributed mainly in Kizilsu (located in Xinjiang province) and the Bohai Rim region. Moreover, regions with higher CAP levels, where the maximum CAP is over 22 days, are distributed sporadically in Henan, Hubei, Hunan, Gansu, and Xinjiang provinces, among which Hetian sees the longest continuous air pollution episode of 123 days with air pollution in the whole year. However, for the average CAP, cities located in the central, southwestern, and northwestern regions have a high average CPA (>6.9 days), while Hetian sees the highest average of a CAP of 23 days, followed by Kashgar’s average of 13.4 days. Notably, a certain number of cities situated in the southeastern, southwestern, and northwestern regions in China, such as Altay, Nyingchi, Lijiang, and Xiameng, show no occurrence of continuous air pollution episodes in 2015, although some of them may experience some air pollution days.

### 3.3. Temporal Variations of Air Pollution

**Figure 4:**
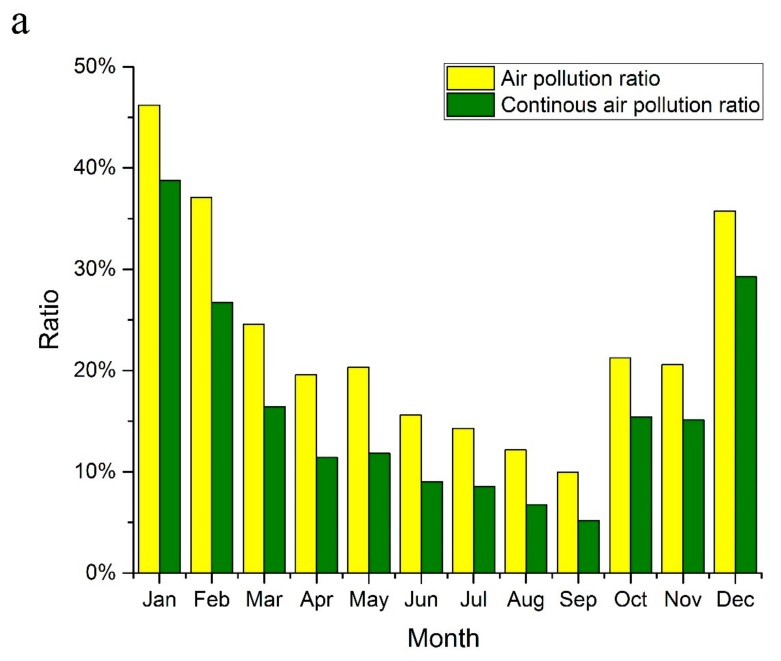

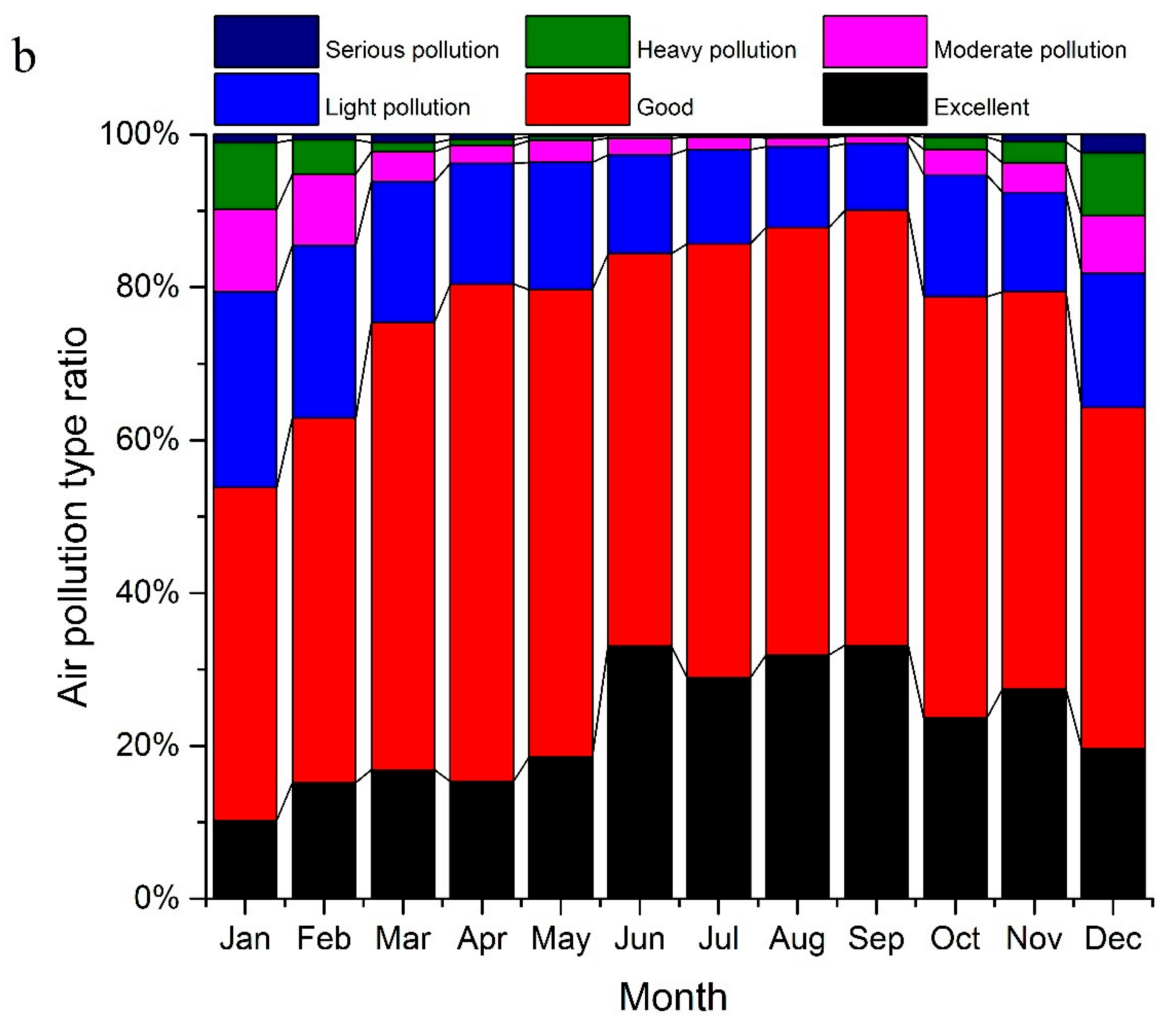
Temporal variations of air pollution in China. (**a**) air pollution ratio and continuous air pollution ratio by month (**b**) air pollution type ratio by month.

Figure 4a shows the monthly temporal variations of air pollution in China. It can be seen that both the air pollution ratio and continuous air pollution (CAP) ratio present a U-shaped variation over the months, with the highest levels in the winter months (December to February) and the lowest levels in the summer months (September to November). Specifically, the highest air pollution ratio and CAP ratio are both observed in January with the values of 46.2% and 38.8%, respectively, while the corresponding lowest values of 10.0% and 5.2% are in September. Due to frequent dust storms in North China that occur mostly in spring, the average air pollution ratio (21.5%) and average CAP ratio (13.2%) in the spring months (March to May) is slightly higher than the corresponding values (17.3% and 11.9%) in the autumn months (September to November). In addition, the coincidence of air pollution ratio and CAP ratio over the months is clearly witnessed in Figure 4a, implying that the occurrence of continuous air pollution episodes is closely associated with the time when air pollution happens. Figure 4b further shows the specific composition of monthly air pollution type. This result suggests that all kinds of air pollution types, ranging from light pollution to serious pollution, are more likely to appear in the winter months, which is especially true for heavy-above air pollution.

### 3.4. Driving Factors of Air Pollution

In this analysis, we take the air pollution ratio as the dependent variable and employ the geographical detector model to test the associations between driving factors and the air pollution ratio. Before running the model, we first transformed the power of determinants (PD) into the *q* statistic in order to distinguish it clearly from the *p*-value commonly used to represent the level of statistical significance.

As shown in Table 3, the third and fourth columns list the results of the *q* statistic and the statistical significance of both natural and human factors in influencing the air pollution ratio using the geographical detector model. The results indicate that all of the natural factors are significantly associated with the air pollution ratio at the 0.05 level. The effect intensities (i.e., *q* statistics) of the natural factors are ranked in descending order as follows: annual average temperature (AAT) > land relief (LR) > relative humidity (RH) > annual average precipitation (AAP) > elevation (ELE) > air pressure (AIRP) > wind speed (WS) > sunshine hours (SH). This result suggests that the air pollution ratio in China is most influenced by annual temperature (with 33.24% of the total variance explained), followed by land relief and relative humidity, which explain 22.94% and 19.10% of the total variance, respectively. With regard to human factors, the significant factors affecting air pollution ratio at the 0.05 level, in descending order, are population density (POPD) > the number of vehicles (NOV) > Gross Domestic Product (GDP) > population (POP) > secondary industry ratio (SIR) > per capita GDP (PGDP) > urbanization rate (UR), indicating that population density, the number of vehicles, and GDP are the three main contributing factors to the air pollution ratio in China, with *q* statistics ranging from 13.30% to 19.46%. Comparing the average of the *q* statistics of the two sets of factors, natural factors, on average, exert greater effects on the air pollution ratio than human factors; the average of effect intensities of natural and human factors on air pollution are 15.95% and 10.37%, respectively.

Nevertheless, although the geographical detector model results exhibited the effect magnitude of each geographical determinant on the air pollution ratio, results from this model have not addressed whether these factors have significant negative or positive effects on air pollution. Therefore, Pearson correlation analysis was further utilized to determine the effect direction of the above influencing factors on the air pollution ratio. The fifth to seventh columns in Table 3 show the correlation coefficients between the driving factors and the air pollution ratio, their corresponding significance, and effect direction. As shown in the table, most of the natural factors, including DEM, land relief, annual average temperature, annual average precipitation, and relative humidity, have significant negative associations with the air pollution ratio in China, while the remaining variables such as wind speed, sunshine hours, and air pressure show inverse relationships with the air pollution ratio. On the other hand, all of the human factors except urbanization rate have significantly positive associations with the air pollution ratio. Their correlation coefficients range from 0.18 to 0.32, indicating that human factors, as proxies of the intensity of human activity, are slightly to moderately associated with air pollution in China. It is, however, remarkable that the result is inconsistent regarding whether urbanization rate is significantly associated with the air pollution ratio in the geographical detector model and Pearson correlation analysis. This result may be explained by the fact that the air pollution ratio is likely to be influenced by urbanization rate within a certain range, within which the variance in the air pollution level may be large, resulting in no correlation with the urbanization rate.

**Figure 5:**
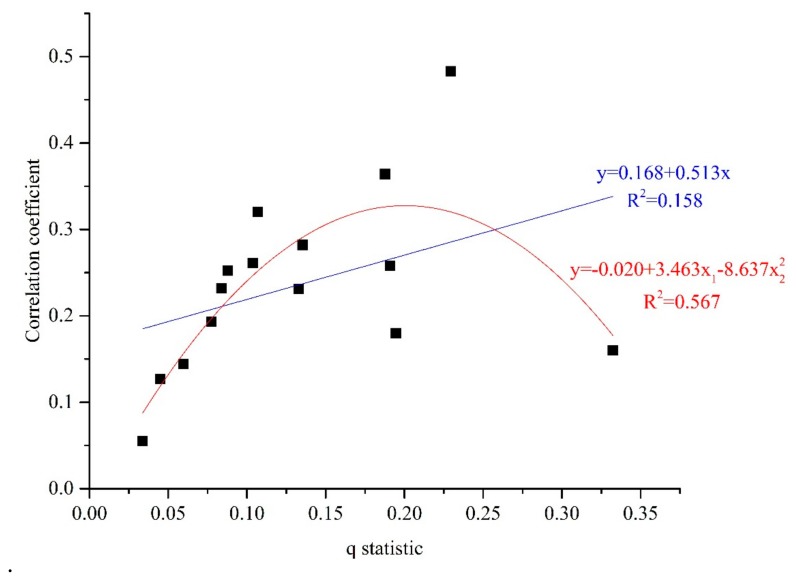
Fitting lines between the *q* statistic and the correlation coefficient.

A linear equation and a quadratic equation were fitted to explore the relationship between the absolute value of the Pearson correlation coefficients and the *q* statistic. Figure 5 shows the fitting results between the *q* statistic and the correlation coefficients. The results reveal that the goodness of fit of the quadratic equation (R^2^ = 0.567) is much better than that of the linear equation (R^2^ = 0.158), which indicates that the relationship between the Pearson correlation coefficients and the *q* statistic fits an inverted U-shaped curve better. This may be explained by the large variance of the air pollution ratio among subcategories of the driving factors such as the annual average temperature, meaning that temperature may have a typical segmentation effect on the air pollution level in China.

## 4. Discussion

To our knowledge, this is the first study using air quality data recorded from ground monitoring stations to identify the spatiotemporal variations of air pollution in Chinese cities above the prefecture level, with a particular focus on continuous air pollution. Furthermore, an innovative approach—the geographical detector model—was used to examine the associations between driving factors and the air pollution ratio, taking into consideration the combined effects of both natural and human-related factors. Our findings provided additional empirical evidence of the spatial and temporal patterns of air pollution in China, helping to identify where air pollution control and mitigation is urgently needed in the future and explain the spatial disparity in air pollution in China.

The results of this study revealed that the average air pollution ratio and continuous air pollution ratio were 23.1% and 16.2%, respectively, and 179 cities (or 46.1%) with an air pollution ratio exceeding 20% did not meet the policy goals. These results suggest that air pollution in China was still serious for the year 2015, although a slightly decreasing trend in the air pollution ratio was seen when compared with the air pollution ratio of 26.6% in 2014 recorded by a recent study [43]. Moreover, the air pollution ratio and the continuous air pollution ratio were much higher in the north, especially in cities located in the Bohai Rim region and Xinjiang province, than in the south, a finding which is consistent with previous studies conducted at the national level in China [18,30,44]. There are several possible explanations for this variation. First, energy consumption in North China depends more on coal burning, which leads to the generation of huge amounts of air pollutants, whereas electricity, the dominant energy source in South China, discharges a smaller volume of air pollutants. Moreover, North China has to provide heating in winter because of the cold weather, and it is economically more dependent on heavy industries compared with South China [18,45], contributing substantially to locally elevated air pollutant concentrations. Another important reason for worse air pollution in the north is the frequent presence of unfavorable climate conditions for air pollutant dispersion and dilution (e.g., weak convection, less precipitation, and frequent occurrences of temperature inversion) [18,44,46].

However, using a complete air quality dataset for 2015 for all Chinese cities above the prefecture level, this study found that air pollution in the western region of China was not lighter than the east, which seems to contradict the results derived from data of an incomplete sample of cities by Lin and Wang [38]. This is probably due to the frequent dust storms in spring which occur mainly in the northwestern region [44]. With regard to the temporal variations of air pollution in China, both the air pollution ratio and the continuous air pollution ratio presented a U-shaped variation over the year, a variation which conforms to the results of a host of previous studies [17,18]. The highest level of air pollution in winter can be explained by at least two reasons [45,47]. One is that home heating during the winter months, especially in the north, gives rise to the increased amount of total coal combustion. Another is that poor meteorological conditions (e.g., temperature inversion and low-powered air convection) in winter relative to summer may hinder the atmospheric dispersion and dilution of air pollutants. Therefore, to control and abate air pollution in China, more efforts should be devoted to addressing the challenges of serious and persistent air pollution episodes that occur mostly during the winter season.

The geographical detector model results indicate that both natural and human factors were significantly associated with the air pollution ratio in China. Negative associations were found between most of the natural factors (i.e., DEM, land relief, annual average temperature, annual average precipitation, and relative humidity) and the air pollution ratio, while positive associations were found between the air pollution ratio and wind speed, sunshine duration, and air pressure, a result which partly aligns with the findings in Luo, Du, Samat, Xia, Che and Xue [33] and Yang et al. [48]. Also, annual average temperature, land relief, and relative humidity have the greatest effects on the air pollution ratio among the natural factors. These findings highlight the significant role of natural factors in the formation process of air pollution through a series of mechanisms, such as providing air pollutants (e.g., sand and dust particles in the desert with dry weather) and changing atmosphere dispersion conditions. For instance, a high temperature is likely to accelerate atmospheric convection and decrease air pollution levels [46], and high wind speed tends to transport air pollutants to down-wind areas and increase air pollution levels (although it may lower air pollution levels of the up-wind areas to some extent) [48]. On the contrary, human factors such as population density, the number of vehicles, GDP, population, secondary industry ratio, and per capita GDP were all found to exert a slight or moderate positive effect on the air pollution ratio in China, a finding in resonance with many previous studies [29,30,33]. Among the human factors, population density, the number of vehicles, and GDP were the dominant factors affecting the air pollution ratio. This result is reasonable because these factors are more related to the intensity of human activities, leading to more fossil energy consumption and pollutant emission.

## 5. Conclusions

Using ground monitoring data of the daily air quality index of Chinese cities above the prefecture level in 2015, this study examined the spatiotemporal patterns of air pollution and continuous air pollution in China. It identified the effect intensity and direction of a set of comprehensive driving factors on the air pollution ratio. Several conclusions are provided as follows. 

Frequent and persistent air pollution episodes in China deserve urgent attention since the air pollution ratio and the continuous air pollution ratio were both high in 2015, with an average of 23.1 ± 16.9% and 16.2 ± 14.8% respectively, and 179 cities (or 46.1%) in China were observed to have an air pollution ratio of higher than 20%, which does not meet the required urban air quality standard of the government. The highest level of the air pollution ratio and the continuous air pollution ratio were located mainly in North China, especially in the Bohai Rim region and Xinjiang province, and the lowest level was found in South China. As for the temporal patterns of air pollution in China, both the air pollution ratio and the continuous air pollution ratio exhibited a U-shaped variation over the year, with the highest values in the winter months and the lowest values in the summer months. Therefore, to improve the air quality in urban China, Chinese governments in the northern region should impose stricter environmental regulations and design adequate emergency measures to address the serious and persistent air pollution episodes that occur mostly in winter months.

Combining the results of the geographical detector model and Pearson correlation analysis, most of the natural factors—including DEM, land relief, annual average temperature, annual average precipitation, and relative humidity, but excepting wind speed, sunshine hours, and air pressure—showed significant negative associations with the air pollution ratio in China, while all the human factors except urbanization rate have significant positive effects on the air pollution ratio. Nonetheless, the effect intensity of natural factors on the air pollution ratio is, on average, greater than that of human factors. More specifically, the air pollution ratio in China was most affected by annual average temperature, land relief, and relative humidity among natural factors, and by population density, the number of vehicles, and GDP among human factors. There are two policy implications from these conclusions. Above all, scientific planning of national industry distribution should take into account the effects of natural factors on air pollution. Reducing dependencies of energy-intensive heavy industries in the north could ameliorate the level of air pollution in view of its disadvantageous natural factors. Furthermore, urban development in China needs to strike a balance between socioeconomic growth and environmental protection in view of the contribution of human activities to the air pollution level. Taking measures such as reasonably evacuating metropolis populations, decreasing the use of vehicles, and transforming economic development modes in China is likely to mitigate air pollutant emissions through reducing the intensity or impact of human activities.

Lastly, this study has several limitations. First, air quality data from ground monitoring stations in this study were only for the year 2015. However, the driving factors and spatial variations of air pollution may vary from year to year. The explanatory power of different driving factors may thus change from year to year and over time at different time scales (e.g., day, week, and year). Future studies should use multiyear data and a multitemporal scale approach. Second, the air quality data used are cross-sectional data and, thus, cannot capture causality accurately. In future research, longitudinal or panel data should be used to explore the causality between driving factors and air pollution. Third, this study only focused on the association between the driving factors and the air pollution ratio, while the effect of the driving factors on continuous air pollution in China was not examined in the quantitative analysis and, thus, needs to be further studied. Lastly, the characteristics of continuous air pollution episodes were neglected in this study. It can be expected that continuous air pollution episodes with all heavy-above air pollution days could generate greater harmful effects than those with light air pollution days.

## Figures and Tables

**Table 1:** Characteristics of air quality index (AQI) and individual AQI (IAQI) concentration limits in China.

AQI Level	AQI Type	Health Effect	SO_2_	NO_2_	PM_10_	CO	O_3_	PM_2.5_
I (0–50)	Excellent	Good	0–50	0–40	0–50	0–2	0–160	0–35
II (51–100)	Good	Moderate	51–150	41–80	51–150	3–4	161–200	36–75
III (101–150)	Light pollution	Unhealthy for Sensitive Groups	151–475	81–180	151–250	5–14	201–300	76–115
IV (151–200)	Moderate pollution	Unhealthy	476–800	181–280	251–350	15–24	301–400	116–150
V (201–300)	Heavy pollution	Very Unhealthy	801–1600	281–565	351–420	25–36	401–800	151–250
VI (301–500)	Serious pollution	Hazardous	1601–2620	566–940	421–600	37–60	801–1200	251–500

Note: The unit for CO is mg/m^3^ while units for the other pollutants are μg/m^3^. IAQI is calculated using the 24 h average concentration of all individual pollutants except for O_3_ which uses the 1 h average concentration.

**Table 2:** Descriptive statistics of air pollution in China.

Measurement Indicators	Average	Std	Median	Min	Max
Air pollution ratio	23.1%	16.9%	21.1%	0.0%	80.7%
Heavy-above air pollution ratio	3.1%	4.4%	1.6%	0.0%	38.1%
CAP ratio	16.2%	14.8%	13.5%	0.0%	76.2%
Times of CAP	10.7	8.8	9.0	0.0	38.0
Maximum of CAP	10.2	9.5	8.0	0.0	123.0
Average of CAP	4.8	2.4	4.6	0.0	23.0

**Table 3:** Results of the effects of driving factors on air pollution.

Drivers	Variables Codes	*q* Statistic	Pearson Correlation Coefficient	Effect Direction
Natural factors	ELE	10.37% **	−0.261 **	−
	LR	22.94% **	−0.483 **	−
	AAT	33.24% **	−0.160 **	−
	AAP	18.77% **	−0.364 **	-
	WS	8.38% **	0.232 **	+
	RH	19.10% **	−0.258 **	−
	SH	5.98% **	0.144 **	+
	AIRP	8.78% **	0.252 **	+
Human factors	POP	10.68% **	0.320 **	+
	POPD	19.46% **	0.180 **	+
	GDP	13.30% **	0.231 **	+
	PGDP	4.49% **	0.127 *	+
	SIR	7.74% **	0.193 **	+
	NOV	13.53% **	0.282 **	+
	UR	3.38% *	0.055	Not significant

Note: “*” and “**” indicate significance at *p* < 0.05 and *p* < 0.01, respectively. As to the variable codes, ELE is elevation, LR is land relief, AAT is annual average temperature, AAP is annual average precipitation, WS is wind speed, RH is relative humidity, SH is sunshine hours, AIRP is air pressure, POP is population, POPD is population density, GDP is Gross Domestic Product, PGDP is per capita GDP, SIR is secondary industry ratio, NOV is the number of vehicles, and UR is urbanization rate.
